# Evaluation of Schlemm’s canal with swept-source optical coherence tomography in primary angle-closure disease

**DOI:** 10.1186/s12886-023-03001-4

**Published:** 2023-06-07

**Authors:** Xuming Ding, Lulu Huang, Cheng Peng, Li Xu, Yixin Liu, Yijie Yang, Ning Wang, Mengyang Gu, Chengyang Sun, Yue Wu, Wenyi Guo

**Affiliations:** 1 Department of Ophthalmology, Ninth People’s Hospital Affiliated to Shanghai Jiao Tong University School of Medicine, Shanghai, 200011 China; 2 Shanghai Key Laboratory of Orbital Diseases and Ocular Oncology, Huangpu District, No. 639 Zhizaoju Road, Shanghai, 200011 China

**Keywords:** Primary angle closure disease, Schlemm’s canal, Iridotrabecular contact, Optical coherence tomography

## Abstract

**Purpose:**

To perform an in vivo evaluation of the changes in Schlemm’s canal (SC) among patients with primary angle-closure disease (PACD) using swept-source optical coherence tomography (SS-OCT).

**Methods:**

Patients diagnosed with PACD who had not undergone surgery were recruited. The SS-OCT quadrants scanned herein included the nasal and temporal sections at 3 and 9 o’clock, respectively. The diameter and cross-sectional area of the SC were measured. A linear mixed-effects model was performed to analyze the effects of parameters on the SC changes. The hypothesis of interest was related to the angle status (iridotrabecular contact, ITC/open angle, OPN), which was further explored with pairwise comparisons of the estimated marginal means (EMMs) of the SC diameter and SC area. In the ITC regions, the relationship between the trabecular-iris contact length (TICL) percentage and SC parameters was also studied by a mixed model.

**Results:**

A total of 49 eyes of 35 patients were included for measurements and analysis. The percentage of observable SCs in the ITC regions was only 58.5% (24/41), whereas it was 86.0% (49/57) in the OPN regions (χ^2^ = 9.44, *p* = 0.002). ITC was significantly associated with a decreasing SC size. The EMMs for the diameter and cross-sectional area of SC at the ITC and OPN regions were 203.34 μm versus 261.41 μm (*p* = 0.006) and 3174.43 μm^2^ versus 5347.63 μm^2^ (*p* = 0.022), respectively. Sex, age, spherical equivalent refraction, intraocular pressure, axial length, extent of angle closure, history of acute attack and treatment with LPI were not significantly associated with SC parameters. In the ITC regions, a larger TICL percentage was significantly associated with a decrease in SC diameter and area (*p* = 0.003 and 0.019, respectively).

**Conclusions:**

The morphologies of SC could be affected by the angle status (ITC/OPN) in patients with PACD, and ITC was significantly associated with a decreasing SC size. These changes in SC as described by OCT scans might help to elucidate the progression mechanisms of PACD.

## Background

Primary angle-closure glaucoma (PACG) is a major ophthalmologic problem worldwide and was estimated to have affected approximately 20 million people in 2020 [1]. PACG is characterized by increased intraocular pressure (IOP) due to occlusion of the trabecular meshwork (TM) by the peripheral iris. Primary angle closure suspect (PACS) and primary angle closure (PAC), which have different levels of iridotrabecular contact (ITC), are predisposing conditions of PACG [2, 3]. Continuous or persistent ITC can lead to the development of peripheral anterior synechiae (PAS), where the iris adheres to the trabecular meshwork, thus causing a gradual closure of the iridocorneal angle [4]. This can result in permanent angle closure and elevation in IOP. Although some patients may not exhibit elevated IOP during ITC, it remains unclear whether there is any structural or functional impairment of the aqueous outflow system during this process.

The SC, which collects the majority of aqueous humor from the anterior chamber and delivers it into the blood vessels, is a circular canal within the perilimbal sclera [5]. Its cross-section is small, thereby hindering its observation. In recent decades, due to technological advances in optical coherence tomography (OCT) – which is a noninvasive, high-speed and high-resolution imaging test – the assessment of human SC in vivo has become possible, and images of SC in heathy eyes have been successfully reported [6]. Studies have demonstrated a decreased SC size in primary open-angle glaucoma (POAG) patients [7] and in elderly people [8]; however, the relationship between SC dimensions and angle closure in PACD eyes remains unclear. Therefore, this study aimed to use swept-source optical coherence tomography (SS-OCT) to evaluate the morphologies of SC in PACD and to discuss the effects of ITC on the canal’s dimensions. In addition, age, sex, refraction, intraocular pressure (IOP) and other factors linked to PACD were also analyzed.

## Methods

### Participants

This was a retrospective observational study that used SS-OCT to examine 40 Chinese subjects diagnosed with PACD who were treated at the Department of Ophthalmology, Shanghai Ninth People’s Hospital. Ethical approval was obtained from the institutional review board of the Shanghai Ninth People’s Hospital affiliated with the Shanghai Jiao Tong University School of Medicine (SH9H-2022-T207-1). Written informed consent was waived.

The following data were collected from medical records: best-corrected visual acuity (BCVA), refractive error, IOP, axial length (AL), slit-lamp examination, fundal examination and gonioscopy. The standard logarithmic visual acuity chart was utilized to evaluate the BCVA. Upon gonioscopy, an angle quadrant was considered “close” if the posterior trabecular meshwork could not be seen in the primary position without indentation (Scheie grade III/IV or modified Shaffer grade 0/I). In addition, the extent of angle closure was recorded for every participant included (Scheie grade: grade I = visible ciliary body, grade II = visible scleral spur, grade III = visible anterior trabecular meshwork, grade IV = angle structures not visible; modified Shaffer grade: grade 0, no structures visible; grade 1, nonpigmented TM visible; grade 2, pigmented TM visible; grade 3, scleral spur visible; and grade 4, ciliary body visible).

Individuals presenting with PACD were enrolled. Stages of angle closure were defined based on the International Society of Geographical and Epidemiological Ophthalmology classification guidelines. As reliable visual fields were not consistently available, PAC and PACG were collapsed into a single category (PAC/PACG) as previously described [9, 10]. Briefly, patients had to meet at least one of the following criteria: (1) Primary angle-closure suspect was defined as having 1 or both eyes with at least 2 quadrants of iridotrabecular contact; (2) subjects were classified as PAC/PACG if, in addition to 2 full quadrants of appositional angle closure, they also had any 1 of the following: IOP > 21 mmHg, peripheral anterior synechia (PAS), a vertical cup-to-disc ratio (C/D) ≥ 0.7, or other evidence of glaucomatous optic neuropathy (notching, nerve fiber layer defects, optic nerve hemorrhage, or vertical cup/disc asymmetry > 0.2); or (3) a history of laser peripheral iridotomy (LPI).

The exclusion criteria were best-corrected visual acuity < 0.1, spherical equivalent refractive error >  + 3 or < -6 D, previous ocular trauma or surgery, and any ocular or systemic conditions that could reduce the OCT image quality of SC.

### Swept-source optical coherence tomography

All participants were examined by a single examiner (DXM) under normal indoor illumination (190 ± 16 Lux) via swept-source OCT (DRI OCT Triton, TOPCON, Japan) using a 1050-nm wavelength, scan speed of 100,000 A-scans per second, and axial resolution of 8 μm. An anterior segment lens unit (AA-1, TOPCON, Japan) was used to examine the iridocorneal angle according to the protocols of Line (H) Anterior seg 3.0 mm mode. The quadrants scanned were the nasal and temporal at 3 and 9 o’clock, respectively. For angle imaging, seated subjects were instructed to stare at an externally fixed light source to ensure that the iridocorneal angle was centered on the instrument’s field of view. Each subfield was scanned three times, and the examiner chose the image with the best quality and least amount of noise. During the examination, the subjects were encouraged to open their eyes as wide as possible on their own without the help of the examiner to avoid additional pressure on the eyes. The superior and inferior quadrants of the eyes were not scanned because pulling the eyelid during these scan acquisitions would be technically inevitable.

### OCT image analysis

**Fig. 1:**
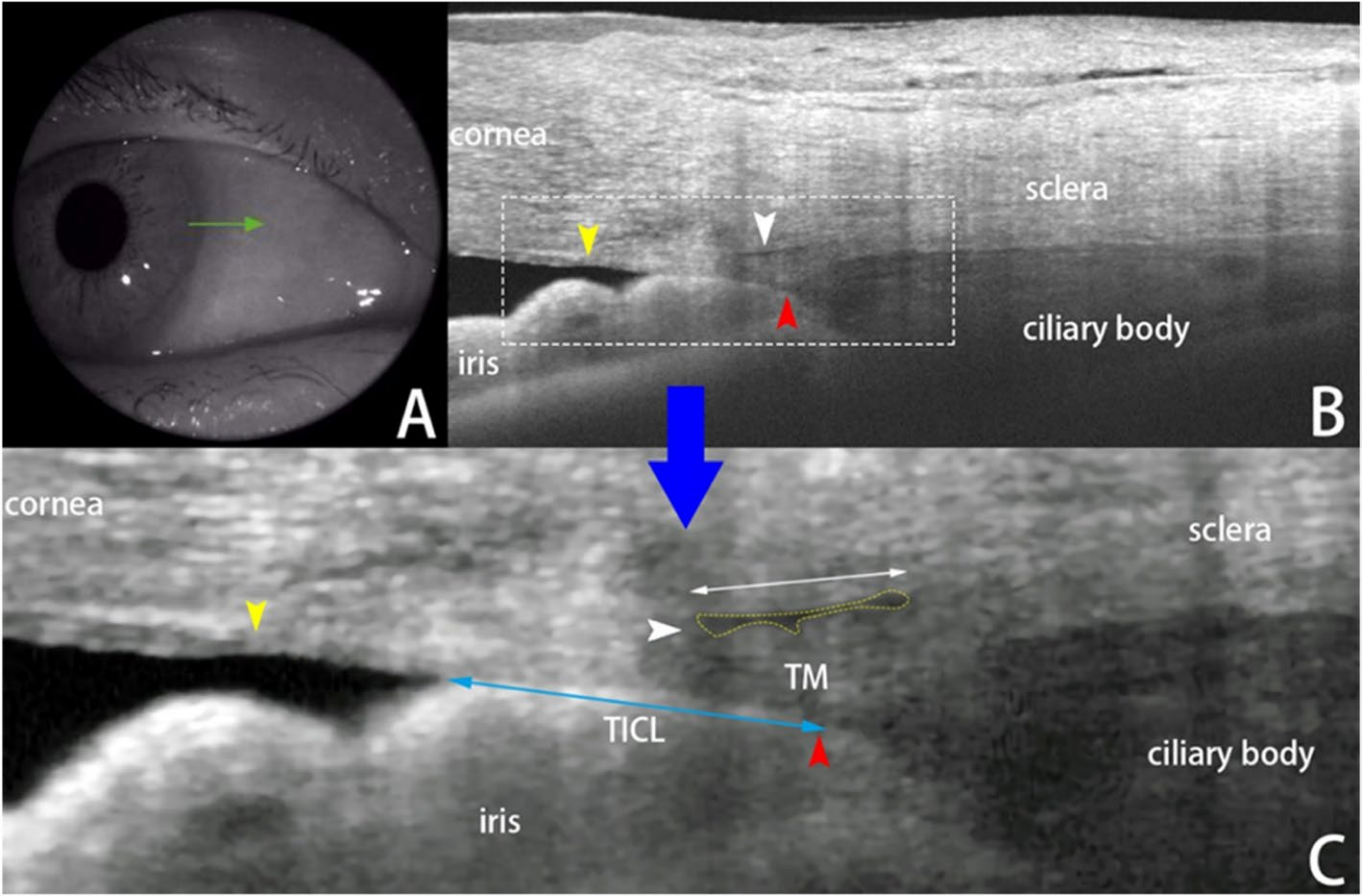
**A** the nasal scan location (3 o’clock) within the right eye was explored by a 3-mm line-scanning model. **B** a SS-OCT B-scan of the nasal iridocorneal angle showing iridotrabecular contact (ITC), the scleral spur (SS, red arrow), Schlemm’s canal (SC, white arrow), the termination of the Descemet membrane (Schwalbe line, SL, yellow arrow) and trabecular meshwork length (TML, distance between SS and SL). **C** the image of **C** was made from the dotted box area in **B** presenting the SC diameter (length of the white double-headed arrow), SC cross-sectional area (SCA, yellow dotted enclosed area), TICL = trabecular-iris contact length (length of the blue double-headed arrow)

All images we selected were exported in JPEG format, and the images were imported into ImageJ software (http://imagej.nih.gov/ij/; provided in the public domain by the National Institutes of Health, Bethesda, MD, USA) to enable measurement. Contrast and bi-thresholding were subjectively adjusted to optimize identification of the structures of interest. According to previous studies, SC was judged as observable when a thin, black, lucent space was found close to the trabecular meshwork, generally outside and posterior to it. The scleral spur (SS) was defined as the point where there was a change in curvature of the inner surface of the angle wall [11]. Schwalbe’s line (SL) was defined as the termination of the Descemet membrane (Fig. 1). A closed angle on the OCT image was defined by the presence of any contact anterior to the SS between the iris and angle wall [12], and the section was called the ITC region. If the image showed an open angle, then we named it the open angle (OPN) region.

For each section where the SC was observable, the SC diameter and cross-sectional area (SCA) were quantified manually. As shown in Fig. 1, the SC diameter was measured from the posterior to anterior SC endpoints. The SCA was drawn by freehand and depicted as the area surrounded by the outline of the SC. In the ITC regions, the TM length (TML, distance between SS and SL) and trabecular-iris contact length (TICL) were also measured to calculate the TICL percentage (TICLp), which was designed to enable accurate ITC quantification [13]. All of these parameters were obtained from three repeated measurements.

The repeatability and reproducibility of the measurements were assessed in all subjects. For interobserver reproducibility, measures were independently made on the images by two masked observers (WY and PC). To determine intraobserver reproducibility, each image was remeasured again in an interval of 1 week by the same observer (WY). If the coefficient of variation (CV) was < 15% and the intraclass correlation coefficient (ICC) was > 0.75, then the measurements were believed to have acceptable intraobserver and interobserver repeatability and reproducibility.

### Statistical analysis

All analyses were performed using SPSS software package version 24.0 (SPSS, Inc., Chicago, IL, USA). Quantitative data for demographic and clinical characteristics are presented as the means ± standard deviations (range: minimum to maximum). To identify the effects associated with SC parameters in PACG eyes, a linear mixed-effects model for repeated measurements was applied with the SC diameter or SCA as a dependent variable. Age, sex, IOP, spherical equivalent refraction (SE), axial length (AL), cup-to-disc ratio (C/D), extent of angle closure, angle status (ITC/OPN), history of acute attack and treatment with LPI were included as the fixed effects. To identify factors related to SC parameters in the ITC regions, a linear mixed-effects model was applied with the SCL or SCA as a dependent variable. Age, sex, IOP, SE, AL, C/D, extent of angle closure, TICLp, history of acute attack and treatment for LPI were included as the fixed effects. The SC parameters were presented as the estimated marginal means (EMMs), as they could give equal weight to each SC section. A chi-squared test was used to analyze the SC detection rates in the ITC and OPN regions. All tests were two-tailed, and significance was defined as *P* < 0.05. Repeatability and reproducibility were analyzed using the CV and the ICC between measurements.

## Results

A total of 54 eyes from 40 PACD patients were initially recruited for this study; however, five participants (5 eyes) were excluded due to poor cooperation or low OCT image quality. The remaining 49 eyes of 35 patients (14 males and 21 females) were included for measurements and analysis of the SC dimensions. The demographic and clinical characteristics of the subjects are displayed in Table 1. The mean age, IOP, SE refraction, AL, anterior chamber depth, and extent of PAS were 61.66 ± 8.65 years (range: 47 to 77 years), 19.1 ± 5.0 mmHg (range: 11.0 to 30.0 mmHg), 0.76 ± 1.15 diopters (range: -3.00 to + 2.88 diopters), 22.77 ± 0.79 mm (range: 21.16 to 24.39 mm), 2.05 ± 0.27 mm (range: 1.55 to 2.53 mm), and 55.44% ± 33.18% (range: 8.33% to 100.00%), respectively. Nine of the eyes had experienced acute primary angle closure, and 11 eyes had previously received treatment for LPI.

**Table 1:** Demographic and clinical characteristics of the study subjects

Parameters	Descriptive Statistics
Age, years	61.66 ± 8.65(47,77)
Gender, male/female	14/21
Spherical equivalent refraction, D	0.76 ± 1.15(-3.00 + 2.88)
BCVA (log MAR)	0.14 ± 0.26(0–1)
IOP, mmHg	19.1 ± 5.0 (11.0,30.0)
Axial length of eye, mm	22.77 ± 0.79(21.16,24.39)
Cup-to-disc ratio	0.56 ± 0.21 (0.30,1.00)
ACD, mm	2.05 ± 0.27 (1.55,2.53)
Extent of PAS	55.44% ± 33.18%(8.33%, 100.00%)
History	
Acute attack, Yes/No	9/40^a^
LPI, Yes/No	11/38^a^
No. of IOP-lowering drugs	
0	16^a^
1	7^a^
2	4^a^
3	15^a^
4	7^a^

Values are presented as the mean ± SD, range (minimum, maximum)

*BCVA* best-corrected visual acuity, *log MAR* logarithm of minimum angle of resolution, log MAR = lg(1/VA), *IOP* intraocular pressure, ACD anterior chamber depth, *PAS* extent of peripheral anterior synechiae, *LPI* laser peripheral iridotomy, *D* diopters

^a^data are the number of eyes

Ninety-eight OCT B-scan images of the iridocorneal angle were acquired; among them, 57 sections were OPN regions, and the rest were ITC regions. The proportion of sections in which the SC was observable was 74.49% (73/98) among all images, 58.5% (24/41) in the ITC regions, and 86.0% (49/57) in the OPN regions (χ^2^ = 9.44, *p* = 0.002). The presence and agreement of PAS and ITC in the nasal and temporal regions are shown in Table 2.

**Table 2:** Presence and agreement of PAS and ITC

Item	Nasal	Temporal
Gonioscopy		
PAS, N	26	18
SS-OCT		
ITC, N	22	19
OPN, N	27	30
Intraclass correlation^a^	0.86	0.49
*p*-value	< 0.001	0.014

*PAS* peripheral anterior synechia, *ITC* iridotrabecular contact, *OPN* open angle

^a^Agreement between PAS and ITC. Type A intraclass correlation coefficients using an absolute agreement definition

**Fig. 2:**
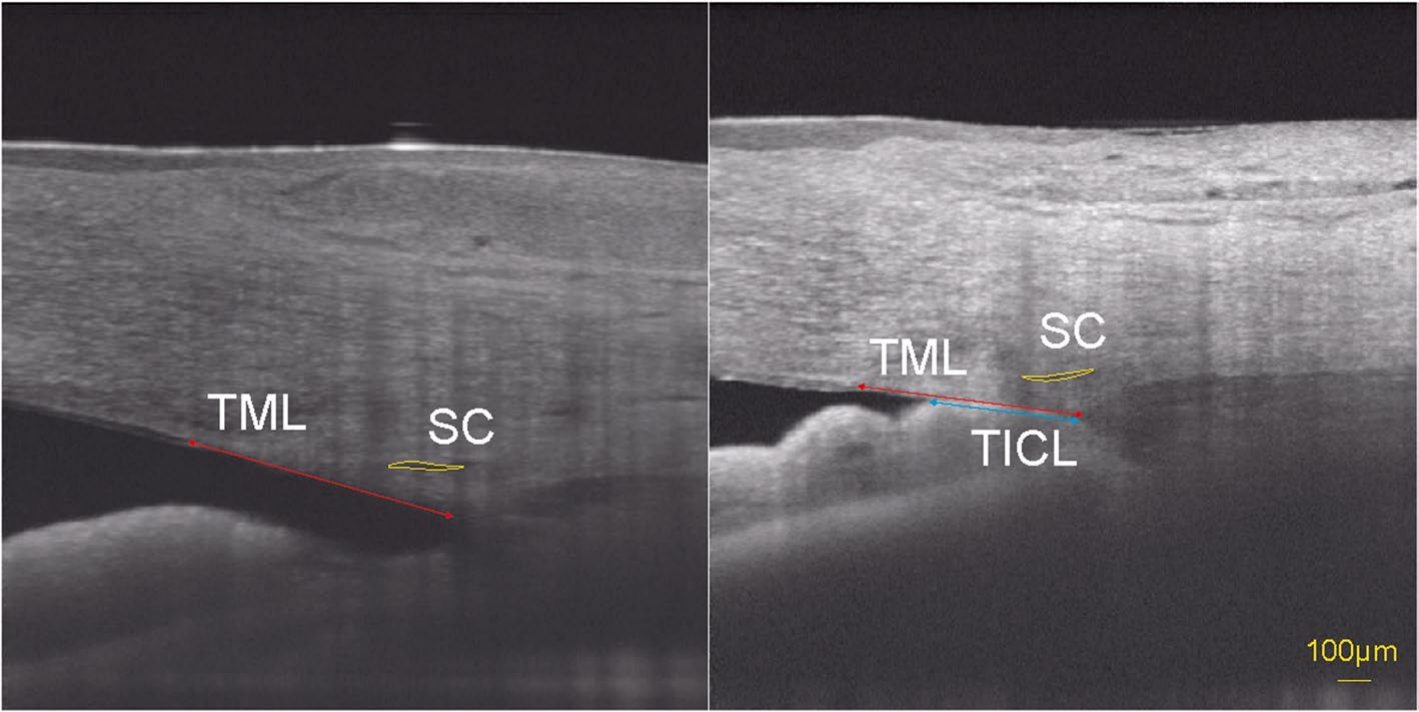
Boundary of SC was sketched out as the yellow curve. TML = trabecular meshwork length (the red double-headed arrow); TICL = trabecular-iris contact length (the blue double-headed arrow)

The results of a linear mixed model analyzing the SC parameters in PACD eyes are shown in Table 3. ITC was associated with decreased SC diameter and SCA. Sex, age, SE, IOP, AL, extent of PAS, history of LPI and acute attack were not significantly associated with the SC parameters. As shown in Table 4, the estimated marginal means (EMMs) at the ITC and OPN regions were 203.34 μm and 261.41 μm (*p* = 0.006) for the SC diameter and 3174.43 μm^2^ and 5347.63 μm^2^ for SCA (*p* = 0.022), respectively. Table 5 further shows that a larger TICL percentage (*p* = 0.003 and 0.019) was significantly associated with a decrease in SCL and SCA in the ITC region. An example is shown in Fig. 2.

**Table 3:** Results of the mixed-effects model analysis to identify factors associated with SC parameters in PACD eyes

	SC diameter (μm)				SC cross-sectional area (μm^2^)			
	EST	*p*-value	95% CI		EST	*p*-value	95% CI	
			Lower	Upper			Lower	Upper
Female	-3.47	0.859	-42.48	35.55	71.39	0.935	-1682.70	1825.48
Age	-1.19	0.215	-3.08	0.71	-40.27	0.347	-125.39	44.85
SE	-0.11	0.989	-16.15	15.92	-304.79	0.400	-1025.61	416.03
IOP	0.78	0.717	-3.53	5.10	40.81	0.675	-153.27	234.88
AL	-14.60	0.258	-40.21	11.00	-839.47	0.149	-1990.56	311.61
PAS extent	-56.08	0.114	-126.11	13.95	110.56	0.944	-3037.97	3259.09
Cup-to-disc ratio	-64.08	0.195	-161.90	33.73	-2878.57	0.195	-7276.27	1519.13
Acute attack	25.95	0.325	-26.38	78.28	350.42	0.766	-2002.20	2703.04
LPI	-39.79	0.129	-91.57	11.99	-1811.81	0.125	-4139.99	516.37
ITC	-58.07	**0.006**	-99.06	-17.08	-2173.20	**0.022**	-4016.05	-330.35

*EST* estimate, *SE* Spherical equivalent refraction, *AL* Axial length, *ITC* iridotrabecular contact, *Acute attack* history of acute attack, *LPI* history of LPI treatment

**Table 4:** Estimated Marginal Means for SC parameters by mixed-effects model

	SC diameter (μm)				SC cross-sectional area (μm^2^)			
	Mean	SE	95% CI		Mean	SE	95% CI	
			Lower	Upper			Lower	Upper
ITC	203.34^a^	21.25	160.73	245.94	3174.43^a^	955.49	1258.78	5090.08
OPN	261.41^a^	13.80	233.74	289.07	5347.63^a^	620.39	4103.81	6591.44
*p*-value^b^	0.006	__	__	__	0.022	__	__	__

*SE* standard error, *95% CI* 95% confidence interval, *ITC* iridotrabecular contact, *OPN* open angle

^a^Covariates appearing in the model were evaluated at the following values: age = 60.82 years, SE = 0.72D, IOP = 18.2 mmHg, AL = 22.76 mm, extent of angle closure = 53.08%, and cup-to-disc ratio = 0.56

^b^Pair-wise comparisons of the estimated marginal means for SC parameters

**Table 5:** Results of the mixed-effects model identifying factors associated with SC parameters in the TIC regions

	SC diameter (μm)				SC cross-section area (μm^2^)			
	EST	*p*-value	95% CI		EST	*p*-value	95% CI	
			Lower	Upper			Lower	Upper
female	1.42	0.941	-36.49	39.32	267.47	0.756	-1451.66	1986.60
age	-1.02	0.281	-2.89	0.86	-34.00	0.425	-118.83	50.84
SE	0.59	0.940	-15.09	16.27	-269.14	0.451	-980.21	441.92
IOP	0.91	0.671	-3.36	5.18	45.56	0.639	-148.06	239.17
AL	-13.72	0.282	-38.99	11.56	-802.78	0.166	-1949.25	343.68
PAS extent	-61.29	0.076	-129.10	6.52	-131.75	0.932	-3207.21	2943.71
Cup-to-disc ratio	-59.11	0.217	-154.04	35.82	-2616.20	0.228	-6921.78	1689.39
Acute attack	33.47	0.199	-18.12	85.05	629.47	0.592	-1710.15	2969.09
LPI	-38.15	0.138	-88.90	12.61	-1720.62	0.140	-4022.66	581.42
TICLp	-76.00	**0.003**	-125.60	-26.39	-2713.64	**0.019**	-4963.46	-463.82

*EST* Estimate, *SE* Spherical equivalent refraction, *AL* Axial length, *Acute attack* history of acute attack, *LPI* history of laser peripheral iridotomy treatment, *TICLp* trabecular-iris contact length percentage

For intraobserver repeatability, the CV and ICC values were 7.5% and 0.95 for the SC diameter, 11.6% and 0.85 for the SC area, 10.4% and 0.88 for the trabecular meshwork length (TML), and 8.8% and 0.90 for the TICL, respectively. For interobserver repeatability, the corresponding CV and ICC values were 12.3% and 0.83 for the SCL, 13.0% and 0.81 for SCA, 9.4% and 0.89 for the TML, and 11.2% and 0.87 for the TICL, respectively.

## Discussion

In this study, we evaluated the changes in SC in patients with PACD in vivo using SS-OCT. We observed that ITC was significantly associated with a decrease in SC size, with a larger TICL percentage correlating with a decrease in SC diameter and area. These results suggest that ITC may play a significant role in the progression of PACD by inducing pathological changes in the SC.

Few histological studies have been conducted to analyze the morphologies of SC in cases of angle-closure glaucoma. Lee described a compressed SC beneath iris adhesions in angle closure glaucoma [14]. Hamanaka suggested that ITC or PAS may be the causal factor in occlusion of the canal [15]. However, to the best of our knowledge, this is the first in vivo study to investigate the relationship between SC parameters and ITC in PACD patients with the help of SS-OCT, which increase the likelihood of accurately presenting the canal's functional size in eyes with PAC.

With the advancement in scan speed and axial resolution, SS-OCT is capable of providing a more detailed and distinct observation of iridocorneal structures [16], and it is becoming a possible method for assessing SC in vivo. A previous study showed that the average SC diameter and cross-sectional area of 50 healthy subjects were 266.96 ± 49.55 μm and 7888.38 ± 1472.58 μm^2^, respectively [17]. Usui et al. examined 60 eyes in vivo in the temporal and nasal sections by SS-OCT, reporting that the percentage of sections in which SC was observable ranged from 85 to 90%, and the average diameter of completely observable SC was 347.2 ± 42.3 μm [18]. Hong and colleagues also described positive detection rates of SC in normal control eyes ranging from 80.0% to 86.0%, with an average SC area of 13991 ± 1357 μm^2^ [7]. Variations in SC dimensions measured across different studies of normal participants may be due to the software for measurement, the population studied, image quality and other factors. In our current study, the SC parameters of different angle statuses (ITC/OPN) were obtained in PACD patients. Our results showed that the SC detection rates and EMMs for the diameter and cross-sectional area of the SC in the OPN region were 86.0%, 261.41 μm and 5347.63 μm^2^, respectively. Although the SC area seemed to be much smaller than that of previous studies in normal eyes, the SC detection rates and diameter were similar to those of healthy individuals. In the ITC regions, the SC detection rates and the EMMs for the SC diameter and area were 58.5%, 203.34 μm and 3174.43 μm^2^, respectively, which were significantly lower than those in the OPN region.

In the present study, we considered that the lower SC identification rates and the reduced SC sizes in the ITC regions might be caused by several reasons. First, ITC typically occurs in the dominant aqueous humor (AH) drainage area of the trabecular meshwork [19]. We have observed the collapse of the Schlemm's Canal in ITC region, suggesting that the AH outflow in the dominant drainage area may also be affected. Second, ITC can also be caused by factors such as pupillary block or plateau iris configuration. In these cases, the iris may push against the trabecular meshwork and SC, leading to SC collapse [20]. Third, SC located in the ITC region might be compressed by the swollen trabecular cells or the thickening extracellular matrix in juxtacanalicular tissue (JCT), which is the outer part of the trabeculum connecting the corneoscleral meshwork with the endothelium of the canal’s inner wall. A light and electron microscopy study by Hamanaka et al. on trabeculectomy specimens from PACG patients showed that the JCT – mostly from the gonioscopically closed area – became compact with swollen trabecular cells, and the majority of the canal was partially or completely occluded [15]. Furthermore, thickening of the amorphous layer beneath the endothelium of the inner wall of the SC was observed in chronic PACG [21]. However, as described by Shihota et al., the trabecular structure in normal eyes was well defined and thin, with many large intertrabecular spaces that could be visualized [22]. Forth, SC might be occluded by melanocytes and pigment-laden trabecular cells. Low-grade mechanical trauma from abrasion between the peripheral iris and the trabecular meshwork may cause infiltration of melanocytes from the iris stoma into the trabecular meshwork [23]. In PACG eyes in which the SC was occluded, the occluded area was replaced by pigment-laden trabecular cells, melanocytes, wide-banded collagen and elastic fibers [15]. In addition, Shihota also found a significant accumulation of pigment granules in the trabecular spaces and Schlemm’s canal in PACG eyes, as seen under both light and electron microscopy [21]. Finally, the SC lumen dimensions are dependent on and reflective of TM motion [24]; hence, we assumed that once the iris is compressed against the inner surface of the meshwork, the SC lumen decreases as a result of TM distention into the SC. These changes could explain the larger SC dimensions in the OPN region compared with those in the ITC region. Furthermore, the fact that SC parameters were associated with ITC but not with the extent of angle closure could also be explained by such changes. Certainly, the mechanical stress caused by the iris on SC remains to be further explored. However, such morphological changes in SC could, to some extent, be the reason for residual glaucoma after LPI, goniosynechialysis or cataract surgery in some PACG patients.

PACG is usually managed by trabeculectomy, trabeculectomy + phacoemulsification, and phacoemulsification + goniosynechialysis (GSL). Phacoemulsification and/or GSL can eliminate ITC areas, but they are unable to address angle dysfunction and resolve SC impairment. Although it has been shown that SC may expand after trabeculectomy, and the degree of this expansion is linked to a decrease in IOP [25], trabeculectomy alone may not be sufficient for more advanced cases with a damaged or even collapsed SC. Furthermore, traditional filtration surgeries are commonly associated with complications. Microinvasive glaucoma surgery (MIGS) is popular in POAG due to its safety and rapid recovery time. Recently, some ophthalmologists have begun to explore the effectiveness of Schlemm’s canal surgery in PACG [26, 27]. As the pathogenesis of POAG and PACG is different, the efficacy of this procedure remains unclear. The results of this study provide further theoretical support for the application of Schlemm’s canal surgery in PACG. In PACG, ITC not only causes lesions in the trabecular meshwork but also alters the morphology of SC. Combined surgery targeting both the anterior chamber angle and SC is expected to manage the outflow resistance of the trabecular meshwork and SC and effectively reduce IOP in PACG patients, provided there is a functional Schlemm’s canal of sufficient size. Liang et al. combined trabeculectomy and tensioning suture-aided Schlemm's canal dilation in PACG patients, which appeared to have good efficacy and safety [26]. Tireh et al. demonstrated that phacoemulsification plus goniosynechialysis and goniotomy was more effective than phacoemulsification plus goniosynechialysis alone in PACG patients [28]. In the current study, the SC size in the ITC region was found to be smaller than that in the OPN region and in healthy eyes in previous studies. Microcatheter-assisted trabeculotomy may be a safer alternative to direct trabeculotomy using instruments such as KDB for treating patients with PACD. Microcatheter-based procedures provide better control and precision during trabecular meshwork dissection, which may reduce the risk of complications associated with direct visualization techniques. Moreover, the use of a microcatheter can make it easier to position and perform the procedure without accidentally damaging the lateral wall of Schlemm's canal or the collector channels due to SC collapse.

Nevertheless, no significant correlation was found between the extent of angle closure and the SC parameters. One possible explanation is that there is no discernible correlation between the position of the ITC and the extent of angle closure. We speculated that ITC is the dominant local factor, and the SC might only be locally obstructed, which could be illustrated by the relatively normal size of SC in the OPN region. In addition, a larger TICL percentage, which was significantly associated with a decrease in SC diameter and SCA (*p* = 0.003 and 0.019), also reflected the extent and severity of local attachment of the iris to the meshwork. Another possible reason is that the results of gonioscopy and OCT imaging do not always agree in angle closure assessments [29, 30]. Tay and his coworkers found that most of the disagreements between gonioscopy and OCT imaging for angle-closure assessments (87.12% of disagreements for AS-OCT, 71.16% of disagreements for SD-OCT) were observed in closed angles (upon gonioscopy) [29]. Sakata et al. reported that angles with a steep "over-the-hill" iris configuration would be described as closed upon gonioscopy but truly open upon OCT imaging [31]. Moreover, mechanical distortion of the cornea during gonioscopy could also possibly make the angles appear artificially narrow [32].

Our study had several limitations. First, we only recruited patients with PACD and did not include any healthy individuals, which would have enabled us to compare the morphologies of SC in the OPN regions between the two groups. Second, the outflow of aqueous humor is not uniform throughout the TM, and it is not equally distributed in the SC and varies between individuals [33, 34]. Furthermore, 3D reconstruction of the SC in vivo has revealed that its diameter is segmental and inconsistent in width [35]. This study only observed two sections of the SC, which may not be representative of the whole canal. However, some studies have demonstrated that there is more angiographic aqueous humor outflow on the nasal hemisphere of the eye [34, 36]. The size of the SC in the nasal and temporal regions may be significant in our study. Further investigations into the variations of the entire SC must be conducted. Additionally, the effects of IOP-lowering drugs on SC size were not evaluated, and they might influence the SC dimensions [37]. The number of intraocular pressure-lowering drugs used in our present study varied from 0 to 4, with active principles including Brimonidine Tartrate, Brinzolamide, Pilocarpine, Carteolol Hydrochloride, Latanoprost, Timolol, and Levobunolol Hydrochloride, thus making it difficult to study the specific drug effects on SC. This highlights the need for further research in this area to investigate the potential role of IOP-lowering drugs in the SC size of PACD. Moreover, our study only included Chinese subjects, which could not rule out the possibility of different racial effects. Furthermore, PAC and PACG were grouped together in a single category, as not all patients had prior visual field examinations. Last, participants were instructed to stare at an externally fixed light during the OCT procedures to expose the quadrant, and we could not verify whether the ocular position would affect the SC measurements. The impacts on ocular positions deserve further examination.

## Conclusions

This is the first in vivo study to evaluate the relationship between SC morphologies and ITC. The morphologies of SC could be affected by the angle status (ITC/OPN) in patients with PACD, and ITC was significantly associated with a decreasing SC size. Such changes in SC observed via OCT scans might enhance our understanding of the progression mechanisms of PACD.

## Data Availability

The datasets used and/or analysed during the current study are available from the corresponding author on reasonable request.

## Abbreviations

AH: Aqueous humor
AL: Axial length
BCVA: Best-corrected visual acuity
C/D: Cup-to-disc ratio
CV: Coefficient of variation
EMMs: Estimated marginal means
ICC: Intraclass correlation coefficient
IOP: Intraocular pressure
ITC: Iridotrabecular contact
LPI: Laser peripheral iridotomy
OCT: Optical coherence tomography
PAC: Primary angle closure
PACD: Primary angle-closure disease
PACG: Primary angle-closure glaucoma
PACS: Primary angle closure suspect
PAS: Peripheral anterior synechia
POAG: Primary open-angle glaucoma
SCA: Schlemm’s canal cross-sectional area
SE: Spherical equivalent refraction
SL: Schwalbe’s line
SS: Scleral spur
TICL: Trabecular-iris contact length
TICLp: Trabecular-iris contact length percentage
TM: Trabecular meshwork
TML: Trabecular meshwork length
