# Correction: Evaluation of Schlemm’s canal with swept-source optical coherence tomography in primary angle-closure disease

**DOI:** 10.1186/s12886-023-03062-5

**Published:** 2023-07-10

**Authors:** Xuming Ding, Lulu Huang, Cheng Peng, Li Xu, Yixin Liu, Yijie Yang, Ning Wang, Mengyang Gu, Chengyang Sun, Yue Wu, Wenyi Guo

**Affiliations:** 1 Department of Ophthalmology, Ninth People’s Hospital Affiliated to Shanghai Jiao Tong University School of Medicine, Shanghai, 200011 China; 2 Shanghai Key Laboratory of Orbital Diseases and Ocular Oncology, Huangpu District, No. 639 Zhizaoju Road, Shanghai, 200011 China

**Correction: BMC Ophthalmol 23, 256 (2023)**

**https://doi.org/10.1186/s12886-023-03001-4**

The original publication of this article [1] contained an incorrect funding section. The incorrect and correct funding information are in this correction article. The original article has been updated.

Incorrect

Clinical Research Program of 9th People’s Hospital afliated to Shanghai Jiao Tong University School of Medicine(No.JYLJ201905).

Correct

Clinical Research Program of 9th People’s Hospital afliated to Shanghai Jiao Tong University School of Medicine(No.JYLJ201904).
